# SENSAAS-Flex: a joint optimization approach for aligning 3D shapes and exploring the molecular conformation space

**DOI:** 10.1093/bioinformatics/btae105

**Published:** 2024-02-21

**Authors:** Hamza Biyuzan, Mohamed-Akram Masrour, Lucas Grandmougin, Frédéric Payan, Dominique Douguet

**Affiliations:** Université Côte d’Azur, CNRS UMR7271, I3S, Sophia Antipolis 06900, France; Université Côte d’Azur, CNRS UMR7271, I3S, Sophia Antipolis 06900, France; Université Côte d’Azur, CNRS UMR7271, I3S, Sophia Antipolis 06900, France; Université Côte d’Azur, CNRS UMR7271, I3S, Sophia Antipolis 06900, France; Université Côte d’Azur, Inserm U1323, CNRS UMR7275, IPMC, Valbonne 06560, France

## Abstract

**Motivation:**

Popular shape-based alignment methods handle molecular flexibility by utilizing conformational ensembles to select the most fitted conformer. However, the initial conformer library generation step is computationally intensive and limiting to the overall alignment process. In this work, we describe a method to perform flexible alignment of two molecular shapes by optimizing the 3D conformation. SENSAAS-Flex, an add-on to the SENSAAS tool, is able to proceed from a limited set of initial conformers through an iterative process where additional conformational optimizations are made at the substructure level and constrained by the target shape.

**Results:**

In self- and cross-alignment experiments, SENSAAS-Flex is able to reproduce the crystal structure geometry of ligands of the AstraZeneca Molecule Overlay Test set and PDBbind refined dataset. Our study shows that the point-based representation of molecular surfaces is appropriate in terms of shape constraint to sample the conformational space and perform flexible molecular alignments.

**Availability and implementation:**

The documentation and source code are available at https://chemoinfo.ipmc.cnrs.fr/Sensaas-flex/sensaas-flex-main.tar.gz

## 1 Introduction

A wide range of two-dimensional (2D) and three-dimensional (3D) methods have been developed to model the shape of molecules and determine their similarities (Kumar and Zhang 2018, Jiang *et al.* 2021). For 3D methods, it is necessary to generate conformers if the experimental structure is not used. Molecular flexibility is a challenging issue because the conformational space is very large and generating conformers could therefore be time-consuming and retrieve a huge number of 3D structures (Hawkins 2017). There are two ways to account for flexibility in 3D methods: using pre-generated conformers (also called conformational ensemble) or exploring the conformational space during the calculation process. The latter can be achieved by searching the torsional space with a fragment-based construction strategy—as in FlexX (Rarey *et al.* 1996) and FlexS (Lemmen *et al.* 1998) or BCL::MolAlign (Brown *et al.* 2019)—or by incorporating a force field to calculate the internal energy of the molecule—as in AutoDock (Morris *et al.* 1998) or POSIT (Kelley *et al.* 2015). Docking and pharmacophore modeling methods use the protein structure and the pharmacophore model, respectively, to constrain the exploration of the conformational space. Early attempts to perform flexible alignment of shapes represented by volume-based representations were addressed by Sastry *et al.* (2011) and Schmidt *et al.* (2018). Phase Shape uses a hybrid approach to refine top alignments of pre-generated conformers to maximize the volume overlap, but does not optimize the input conformation (Sastry *et al.* 2011). ReFlex3D aims to refine the conformation, but it requires starting with a very large number of initial conformers (Schmidt *et al.* 2018). Here, we present a surface-based method that allows to jointly optimize 3D shape alignment and explore molecular conformational space.

Recently, we described a method called SENSAAS that uses dense and colored 3D point clouds to represent the molecular shape (Douguet and Payan 2020). SENSAAS performs rigid molecular alignments using point-based representation of the van der Waals surface to provide superimposition of 3D graphs and similarity scores. A specificity of SENSAAS is that it optimizes the superimposition of two molecules by matching similar exposing areas in terms of geometry and properties, instead of maximizing the volume overlap as do methods using atom-centered Gaussian functions (Grant *et al.* 1996, Vainio *et al.* 2009, Liu *et al.* 2011). Hence, SENSAAS is able to identify local similarities and align substructures or small fragments on large molecules and thus is suited for scaffold hopping and bioisosteric replacement (Douguet and Payan 2020). Our results also showed that SENSAAS was able to reproduce the AstraZeneca Molecule Overlay Test set (AZ dataset) (Giangreco *et al.* 2014) of dissimilar ligands that bind to the same protein with an accuracy comparable to that of popular methods using atom-centered Gaussian functions, while outperforming them in finding more accurate alignments in the interval of root-mean-square distance (RMSD) between 0 and 0.5 Å (Douguet and Payan 2020). However, under real-life conditions, the experimental or bioactive 3D conformation of a molecule, and therefore its shape, is not always known when one wants to compare two molecular structures. In the course of our project, we wanted to be able to deal with conformational flexibility, but without having to generate a large number of conformations.

In the present work, we introduce SENSAAS-Flex, an add-on to SENSAAS, to perform flexible alignment of two point-based shapes without drastically increasing the running time. Our algorithm splits molecular surfaces and 3D graphs to locally optimize the alignment of molecular fragments and sub-shapes. Utilizing 3D graphs remains essential because computational methods require this molecular representation to generate conformers, minimize the conformational energy, calculate properties, or for visualization. We report two benchmark studies where the objective is to reproduce the crystal structure geometry: a self-alignment experiment in which a small set of conformers is aligned on the experimental X-ray structure used as shape reference and a cross-alignment experiment in which a small set of conformers is superimposed to the experimental shape of other ligands that bind to the same protein. We evaluated the performance of rigid and flexible alignments provided, respectively, by SENSAAS and SENSAAS-Flex, and compared it to that of other published methods applied to the AZ data.

## 2 Materials and methods

### 2.1 Rigid alignment with SENSAAS

**Figure 1.**
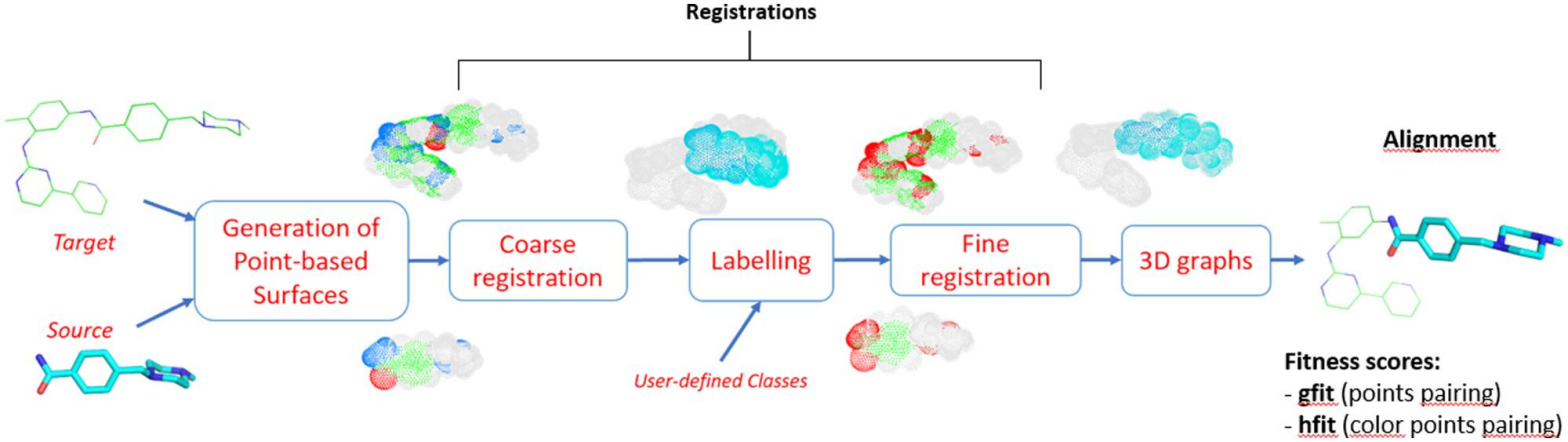
SENSAAS workflow. Here, the Target drug Imatinib (in green) and one of its substructure (Source in cyan) are aligned and scored. Imatinib and its substructure are represented by a point cloud of 6387 and 3668 colored points, respectively. The color of classes shows pharmacophore features: lipophilic (class 1 colored in white/grey), polar (class 2 colored in red), and aromatic (class 3 colored in green) groups.

Let us consider two molecules, called Source and Target, that we want to superimpose to calculate their similarities. SENSAAS (SENsitive Surface As A Shape) aligns their shape by using the 3D point-based representation of their van der Waals surface (Douguet and Payan 2020). SENSAAS follows five major steps, depicted in Fig. 1: (i) generation of a 3D point cloud representing the molecular surface of each input molecule; (ii) coarse alignment of the two point clouds thanks to a geometry-aware global registration (Rusu *et al.* 2009); (iii) labeling of each point of the two point clouds according to the pharmacophoric class of its closest atom [Class 1 includes nonpolar hydrogen and halogen atoms excepting fluorines. Atoms in class 1 contribute the most to the surface geometry and coloration and depict the apolar surface area. Class 2 includes polar atoms able to be involved in hydrogen bonds such as N, O, S, F, and H (if linked to N or O). Class 3 includes “skeleton elements” such as C, P, and B. Class 4 includes all elements not listed in the first three classes. This class is empty for most small organic molecules in medicinal chemistry]; (iv) refinement of the alignment by applying a color and geometry-aware registration (Park *et al.* 2017); (v) Computation of fitness scores which are similar to Tversky coefficients where α = 0. Here, the fitness scores of the Source equals 1.0 (out of 1.0) if its shape is perfectly embedded in the shape of the Target. The fitness score *gfit* evaluates the similarity of shapes in term of geometry only. The fitness score *hfit* evaluates the similarity of the overlapping points having the same property except for the nonpolar hydrogen class. The output is a transformation matrix T (rotation and translation), that allows the alignment of the Source on the Target which is kept fixed in the 3D space. Source code in python and documentation of SENSAAS are available on GitHub (https://github.com/SENSAAS/sensaas-py).

### 2.2 Flexible alignment with SENSAAS-Flex

**Figure 2.**
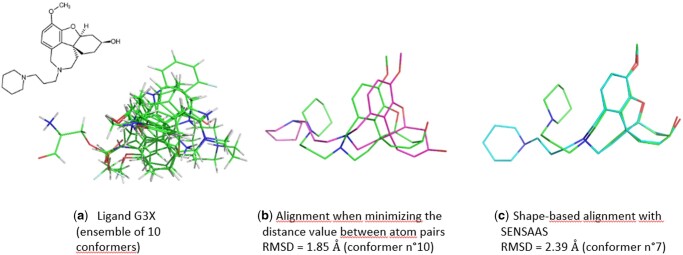
SENSAAS favors the perfect alignment of a substructure instead of a rough alignment. (a) An ensemble of ten conformers of the ligand G3X (PDB 3I6M) was generated using RDKit. Superimposition of 3D graphs and RMSD value between the experimental conformation (in green) and the closest conformer (in magenta) when (b) 3D graphs are aligned with the program SymmFit (Section 2), (c) shapes are aligned using SENSAAS [RMSD value calculated ‘in place’ using CalcLigRMSD (Section 2)]. The RMSD value is larger in the latter case, but the substructures are aligned. PyMOL version 1.3 was used to generate illustrations.

Contrary to methods minimizing the RMSD between atom pairs that favor a rough molecular alignment, SENSAAS is particularly efficient in finding and aligning substructures that perfectly match while leaving the rest of the structure misaligned (Fig. 2).

**Figure 3.**
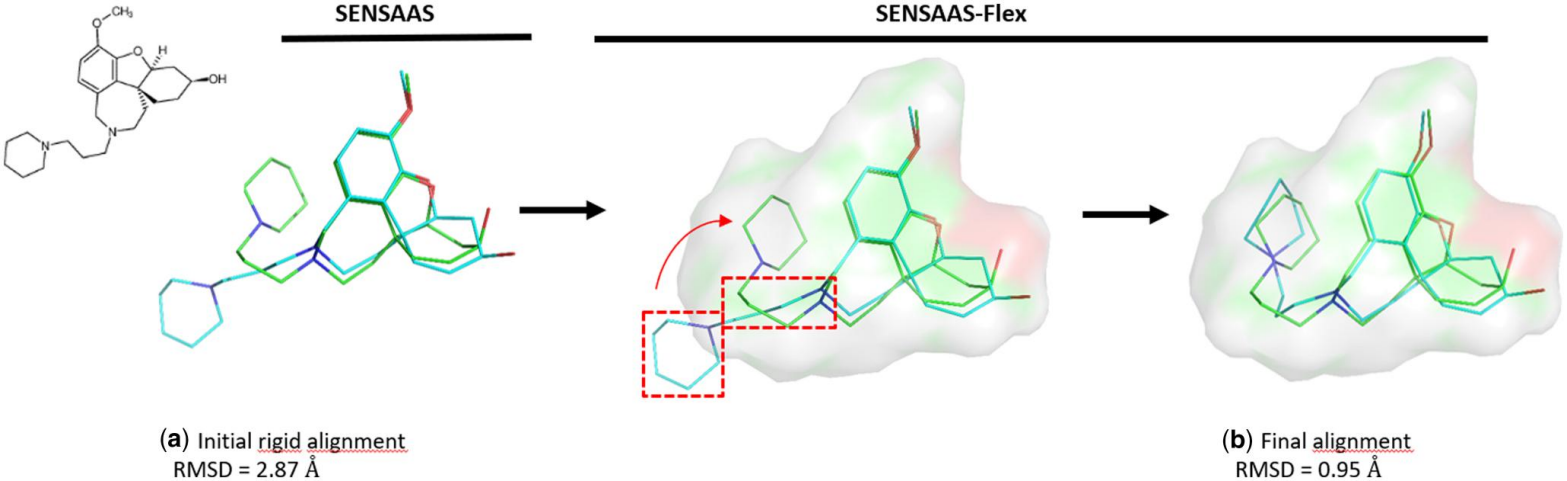
SENSAAS-Flex is able to identify a conformer that is closer to the experimental one. It improves an initial rigid alignment by optimizing the position of misaligned fragments (red insets) according to the neighboring Target shape (for visualization purpose, a solid surface is displayed instead of the 3D point-based representation of the Target shape). Example with a conformer of the ligand G3X (PDB 3I6M) generated using RDKit (in cyan) and its experimental conformation (in green). A RMSD value of 0.95 Å is obtained after the optimization of the conformer. The conformational energy of the X-ray conformation, the initial conformer and the optimized conformer are 250, 135, and 157 kcal/mol, respectively. PyMOL version 1.3 was used to generate illustrations.

SENSAAS-flex relies on this singularity. Starting with a rigid alignment proposed by SENSAAS, SENSAAS-Flex proceeds as follows to perform a flexible alignment: (i) the Source molecule is fragmented according to the work of Bemis and Murcko (1996) (i.e. ring systems, linkers, and substituents); (ii) fragments are independently re-aligned with the neighboring Target point cloud to try to improve their individual fitness scores; (iii) the fragments are reassembled and the structure minimized using the MMF94 force field implemented in RDKit because bond lengths and bond angles may deviate from optimal geometries. As fragments are locally repositioned, a conformational minimization step is likely to find a conformation close to the input and with low conformational energy; (iv) the resulting conformer is aligned one last time on the Target using the color and geometry-aware registration step of SENSAAS; (v) The resulting alignment is only kept if the *gfit* + *hfit* fitness score is better than that of the initial rigid overlay. Figure 3 and Supplementary Fig. S1 show successful flexible alignments with a small and large molecule, respectively.

### 2.3 Preparation of ligands of the benchmarking datasets

We used two benchmark datasets: the AstraZeneca Molecule Overlay Test set (AZ dataset) (Giangreco *et al.* 2013, 2014) and the PDBbind refined dataset (2020) (Liu *et al.* 2017).

The AZ dataset contains overlays of dissimilar ligands that bind to the same protein. We used this dataset in self- and cross-alignment experiments. Originally created for the validation of pharmacophore programs, the AZ dataset is also suitable for evaluating the ability of a shape-based algorithm to reproduce the crystal structure geometry of the overlay. The AZ dataset consists of 121 diverse proteins co-crystallized with 1465 ligands. Giangreco *et al.* (2014) calculated the Borda tallies for three parameters (shape matching, color score, and Tanimoto coefficient used to measure the 2D fingerprint similarity), and ranked them in ascending order. It resulted in four distinct groups, on the basis of how easy or difficult it would be for a program to reproduce them. The overlays are grouped by increasing difficulties: 22 easy (185 ligands), 73 moderate (991 ligands), 18 hard (190 ligands), and 8 unfeasible (99 ligands) sets. Of note, the unfeasible category contains overlays with ligands that are not superimposed, i.e. they bind at different sites of the binding pocket (Supplementary Fig. S2). According to our fragmentation protocol, the average number of fragments per molecule is 3.82 (ranging from 1 to 9). We encountered problems with the structure-data file (SDF) file of six ligands: P00929-3.sdf, P09955-4.sdf, P43235-1.sdf, Q00511-3.sdf, Q16539-8.sdf, and P27487-12.sdf. The first four ligands were modified by removing the “M CHG” line and by setting the atom block related to the charge of the oxygen of the carboxylate function to 0 because a charge of −1 was assigned to the double bonded oxygen in the original SDF file. The ligand Q16539-8.sdf was modified by removing the “M CHG” line and by setting the atom block related to the charge of the oxygen of the pyridinyloxy to 0 instead of 5. The ligand P27487-12.sdf was modified by removing an extra bond between atom 48 and 22. Additionally, the first molecule in the set A9JQL9 was separated into two SDF files (A9JQL9-1–1.sdf and A9JQL9-1–2.sdf) because the two ligands are not covalently attached.

The PDBbind refined dataset of 2020 was downloaded from Zenodo https://zenodo.org/record/5469978. This set contains a total of 5316 ligands extracted from good quality protein-ligand complexes selected from the Protein Data Bank (PDB) (Liu *et al.* 2017). We used this dataset in self-alignment experiments to assess the performance of SENSAAS-Flex on a dataset larger than the AZ one (only 285 common PDB structures). According to our fragmentation protocol, the average number of fragments per molecule is 3.94 (ranging from 1 to 14).

### 2.4 Generating conformers and evaluating structural deviations

The conformer generator RDKit (Open-Source Cheminformatics Software, Release_2019_03_4) (Riniker and Landrum 2015) was used with the following options: input co-crystallized structures are read unmodified (removeHs = False), the experimental-torsion basic knowledge distance geometry (ETKDG) method is selected to generate conformers, filtered with the option pruneRmsThresh = 1 and optimized by the MMFF94 force field. The ETKDG method also addresses ring conformations.

The second tested conformer generator was the knowledge-based program CORINA (Molecular Networks GmbH) (Gasteiger *et al.* 1990).

The MMFF94 force field implemented in RDKit is used to minimize the energy of conformers after SENSAAS-Flex reassembles the re-aligned fragments. In our implementation, we run 500 minimization cycles to generate a low-energy conformer.

The structural deviation is defined as RMSD which sums the distance between corresponding atom pairs in 3D graphs. The RMSD value was calculated using two RDKit based tools. The first one is SymmFit (author: Paolo Tosco; code is available at https://www.mail-archive.com/rdkit-discuss@lists.sourceforge.net/msg04915.html). It allows the minimization of RMSD value but only when the atoms in the two structure files are arranged in the same order. The second one is CalcLigRMSD (author: Carmen Esposito; code is available at https://github.com/cespos/rdkit/tree/add-CalcLigRMSD-for-prealigned-compounds/Contrib/CalcLigRMSD). It allows the calculation of RMSD value of “in place” structures (no minimization) whatever the arrangement of atoms in the two structure files.

### 2.5 RMSD distributions of initial conformers

**Figure 4.**
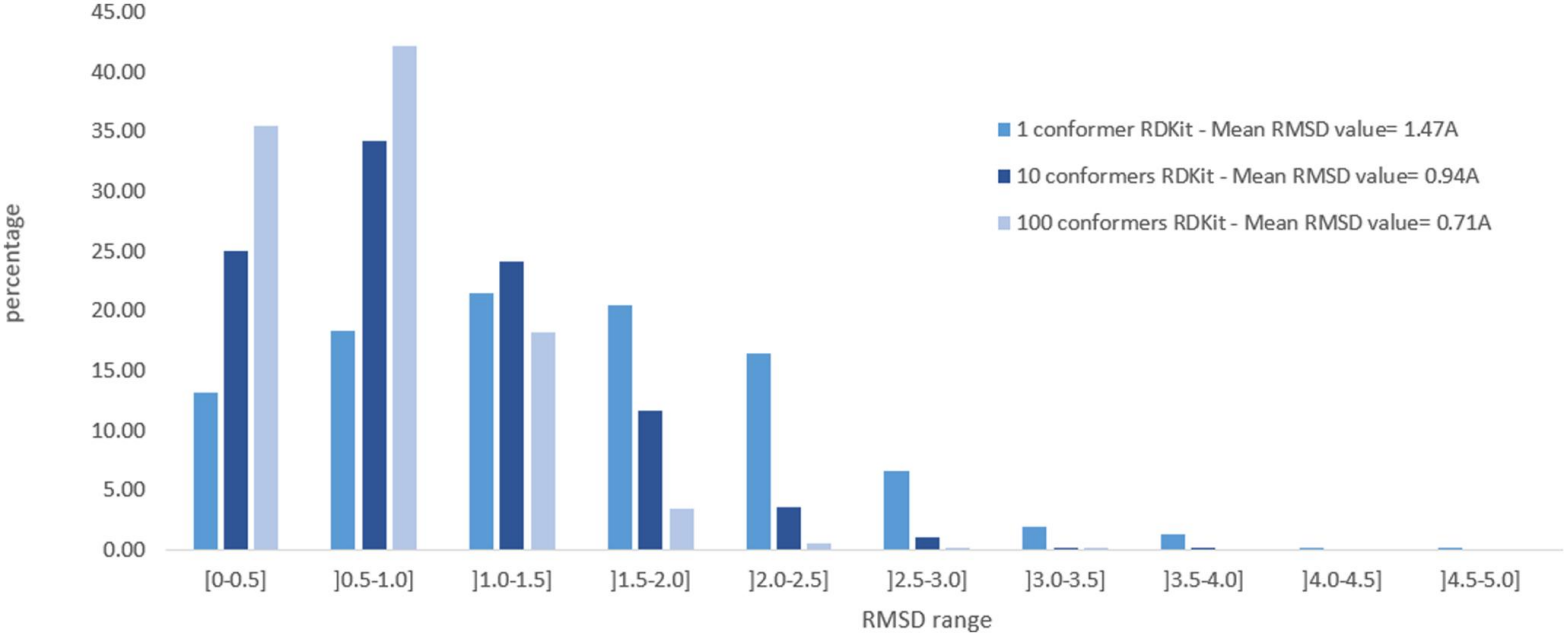
Distribution of RMSD values between the closest conformer and the experimental conformation in the AZ dataset in function of the number of conformers generated with RDKit.

The distribution of RMSD values between co-crystallized structures and conformers was calculated for molecules of the AZ dataset using the program SymmFit. Figure 4 shows that the probability to generate a conformer close to the experimental conformation—in the range [0–0.5]—is larger when one hundred conformers are requested. In Supplementary Fig. S3, we also show that increasing the number of conformers leads to decreasing the proportion of conformers with an RMSD value in the range [0–0.5] and ]0.5–1.0]. Therefore, increasing the number of conformers helps to generate closer conformers to the experimental conformation but those are more diluted in the ensemble of conformers. In addition, Supplementary Figs S4 and S5 show that when 10 conformers are requested, the RMSD distributions for the closest conformer and all conformers, respectively, are similar for the ensemble of conformers generated by RDKit or CORINA (for the AZ dataset) or for the PDBbind refined dataset.

For the PDBbind refined dataset, the distributions for the closest conformer and all conformers are more widespread (mean RMSD value of 1.17 Å and 1.98 Å) than for the AZ dataset (mean RMSD value of 0.94 Å and 1.67 Å). This is due to their difference in size and chemical diversity.

### 2.6 Computing time

All experiments were carried out on a Dell PowerEdge R940 (Intel Xeon Gold 5220) in Linux environment (Debian distribution, version 5.10.46). The processing time of a rigid alignment is about 6 s, and the processing time of an additional flexible alignment is about 7 s per fragment. Therefore, in the latter case, it takes about 28 s to align a molecule composed of four fragments using SENSAAS-Flex.

## 3 Results

### 3.1 Self-alignment experiments

In these experiments, we work with ligands for which we have an experimental conformation used as shape reference (extracted from the co-crystal structure of a complex with a protein), and an ensemble of generated conformers. In self-alignments, we expect the alignment method able to identify a conformer that will perfectly or, close-to-perfectly, be superimposed to the experimental conformation. Furthermore, it is straightforward to evaluate self-alignments by calculating the RMSD which sums the distance between corresponding atom pairs in 3D graphs.

The first experiments were to determine the number of starting conformers and repeats (also called runs) required by SENSAAS-Flex to reproduce the crystal structure geometry of ligands in a reasonable computation time. Because SENSAAS and SENSAAS-Flex belong to stochastic methods as do other computational tools like docking programs, several runs can produce different alignments especially when several local optima exist. Increasing the number of runs can therefore improve performance. Here, in the case of several runs, only the best scored alignment was selected. In parallel, increasing the number of starting conformers allows many initializations. We expect to get good results if the molecular conformation space is sufficiently explored.

Rigid and flexible alignments were evaluated by calculating the RMSD value between the aligned conformer (also called pose) and the experimental X-ray conformer. The lower the RMSD value, the better the superimposition and the closer the structures. A pose is considered as perfect if the RMSD value equals 0 Å, as very good if the RMSD value is in the range ]0–0.5] Å, as good if the RMSD value is in the range ]0.5–1.0] Å, and as acceptable if the RMSD value is in the range ]1.0–2.0] Å.

A series of experiments was carried out varying the number of conformers in the ensemble of conformers (1, 2, 5, 10, or 100) and runs (1, 2, or 5). Table 1 presents the mean RMSD values obtained on the AZ dataset with SENSAAS and SENSAAS-Flex for different number of conformers and runs. The best mean RMSD value of 0.64 Å was obtained with SENSAAS-Flex when using 10 conformers and two runs; the latter are now considered to be the default settings. The experiment using 5 conformers and 5 runs led to slightly lower results (mean RMSD value of 0.68 Å) despite 25 initializations (5 × 5). This result suggests that it is more efficient to start with more conformers (i.e. 10) and only repeat the alignment 2 times (i.e. 20 initializations). When comparing with SENSAAS, it is now evident that even 100 conformers are not enough to achieve the results of a flexible alignment. As the computation time exceeds that of the flexible calculation when 100 conformers are rigidly aligned, we have not attempted to test larger sets of conformers.

**Table 1.** Self-alignment experiments using SENSAAS and SENSAAS-Flex—Rigid and flexible alignment results in function of the number of starting conformations and runs.^a^

	AZ dataset									
Method	Rigid	Rigid	Flexible	Flexible	Flexible	Flexible	Flexible	Flexible	Flexible	Flexible
Nb conformers	10	100	1	1	1	5	5	5	10	10
Run(s)	2	2	1	2	5	1	2	5	1	2
Mean RMSD	1.77	1.4	2.58	1.6	1.18	0.96	0.81	0.68	0.75	**0.64**
RMSD range:										
[0–0.5] Å (%)	18.4	22.8	23.9	27.6	33.3	41.5	47.9	53.5	50.3	**55.8**
[0–1] Å (%)	39.2	49.5	46.2	53.9	62.4	72.2	78.2	83.4	80.2	**85.2**
[0–2] Å (%)	68.3	78.1	70.7	79.7	86.1	90.9	94.2	96.7	94.9	**97.3**

a The mean RMSD value and percentage of conformations in three ranges [0–0.5] Å, [0–1] Å, and [1–2] Å are indicated for each experiment condition. The best values are in bold.

Table 1 and Supplementary Fig. S6 also indicate the percentage of conformations in three RMSD ranges: [0–0.5] Å, [0–1.0] Å, and [0–2.0] Å. Interestingly, SENSAAS-Flex succeeds in enriching the three RMSD ranges. The percentage of conformations in the more precise range [0–0.5] Å is particularly improved [55.8% for SENSAAS-Flex against 22.8% for SENSAAS (100 conformers and 2 runs)]. Similarly, the percentage of conformations in the range [0–1.0] Å and [0–2.0] Å are 85.2% and 97.3% for SENSAAS-Flex and 49.5% and 78.1% for SENSAAS (100 conformers and 2 runs), respectively.

**Figure 5.**
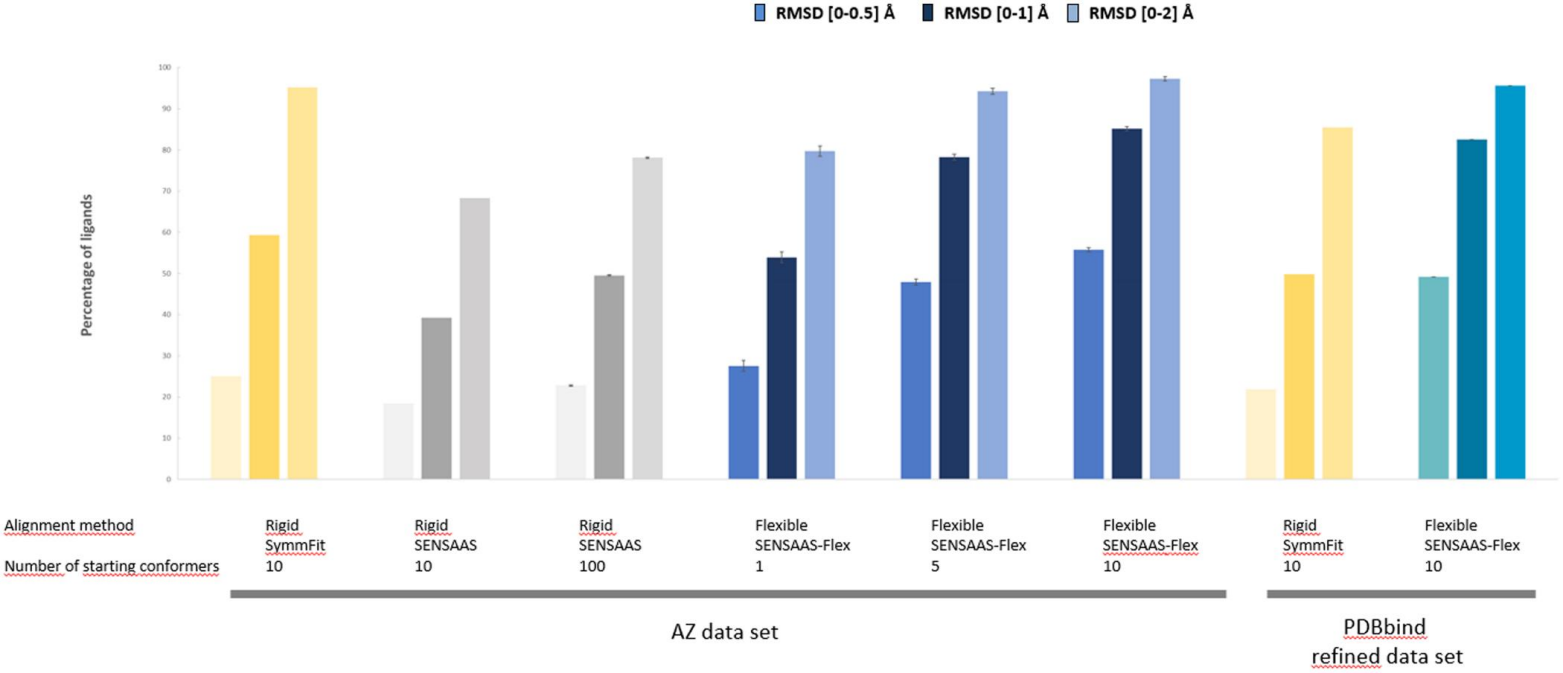
Self-alignment experiments—Rigid and flexible alignment results in function of the method, number of starting conformations and runs, and the dataset. Histograms indicate the percentage of ligands showing a structural deviation between the aligned conformer and the experimental X-ray structure of less than 0.5 Å (first bar), 1 Å (second bar), and 2 Å (third bar). A standard deviation was calculated using five experiments.

We then benchmarked SENSAAS-Flex on the PDBbind refined dataset with the default settings, to ensure that the method is still performing well on a larger and more diverse dataset. Table 2 and Fig. 5 show that comparable mean RMSD value is obtained with SENSAAS-Flex on the PDBbind dataset (0.69 Å), indicating that the default settings are appropriate. Under those conditions, 49.2% of poses show a structural deviation of less than 0.5 Å, 82.5% of less than 1 Å, and 95.6% of less than 2 Å.

**Table 2.** Self-alignment experiments—Rigid and flexible alignment results in function of the method, number of starting conformations and runs, and the dataset.^a^

	AZ dataset						PDBbind refined dataset	
Method	Rigid SymmFit	Rigid SENSAAS	Rigid SENSAAS	Flexible SENSAAS-Flex	Flexible SENSAAS-Flex	Flexible SENSAAS-Flex	Rigid SymmFit	Flexible SENSAAS-Flex
Nb conformers	10	10	100	1	5	10	10	10
Run(s)	1	2	2	2	2	2	1	2
Mean RMSD	0.94	1.77	1.4	1.6	0.81	**0.64**	1.17	**0.69**
RMSD range:								
[0–0.5] Å (%)	25.0	18.4	22.8	27.6	47.9	**55.8**	21.9	**49.2**
[0–1] Å (%)	59.3	39.2	49.5	53.9	78.2	**85.2**	49.8	**82.5**
[0–2] Å (%)	95.1	68.3	78.1	79.7	94.2	**97.3**	85.5	**95.6**

a The mean RMSD value and percentage of conformations in three ranges [0–0.5] Å, [0–1] Å, and [0–2] Å are indicated for each experiment condition. The best values are in bold.

Table 2 and Fig. 5 also present the performance of the program SymmFit when it minimizes the RMSD between atom pairs. As expected, the percentage of conformations for the AZ dataset in the range [0–0.5] Å (25%), [0–1] Å (59.3%), and [0–2] Å (95.1%) with SymmFit are lower than those obtained with SENSAAS-Flex (55.8%, 85.2%, and 97.3%, respectively). This difference is also observed for the PDBbind dataset: 21.9%, 49.8%, and 85.5% with SymmFit and 49.2%, 82.5%, and 95.6% with SENSAAS-Flex.

In Supplementary Table S1, we report RMSD values when CORINA is used to generate conformers for the AZ dataset. Comparable but slightly worse results are achieved with a mean RMSD value of 0.76 Å instead of 0.64 Å. Those lower results can be explained by the fact that CORINA does not always generate the 10 requested conformers. CORINA is a knowledge-based generator that does not sample the molecular conformation space but generates low-energy conformations for each input structure. The total number of conformers for the AZ dataset using RDKit and CORINA are 10 550 and 5943, respectively.

Therefore, the lower number of conformers may explain a deterioration in the results. However, they remain very good.

In conclusion, results of the self-alignment experiments show that SENSAAS-Flex successfully reproduces most of the crystal structure geometry of ligands even when a small set of initial conformers is used. In Supplementary Fig. S7, we further show that there is no correlation between the initial RMSD value and the final one. Interestingly, the closest conformation from the ensemble of conformers does not necessarily grant access to the best final conformer. We calculated that 48% of conformers that lead to the final solution are not the closest conformer in the ensemble (RMSD difference ≥ 0.2 Å).

### 3.2 Cross-alignment experiments

Cross-alignment experiments consist in assessing the ability of SENSAAS and SENSAAS-Flex to reproduce the crystal structure geometry of dissimilar ligands that bind to the same protein (overlay). Such experiments have proven to be much more difficult than self-alignments, but they allow a more accurate assessment of the performance of an alignment method in use cases. Here, conformer(s) of each ligand were superimposed to the experimental shape of the others. Then, the best predicted ensemble is generated by selecting the best aligned conformer of each ligand based on its lowest RMSD with its experimental geometry. Cross-alignment experiments were performed on the AZ dataset. This benchmarking dataset contains 121 diverse proteins co-crystallized with 1465 ligands in total. The overlays are grouped by increasing difficulties: 22 easy (185 ligands), 73 moderate (991 ligands), 18 hard (190 ligands), and 8 unfeasible (99 ligands) sets.

**Figure 6.**
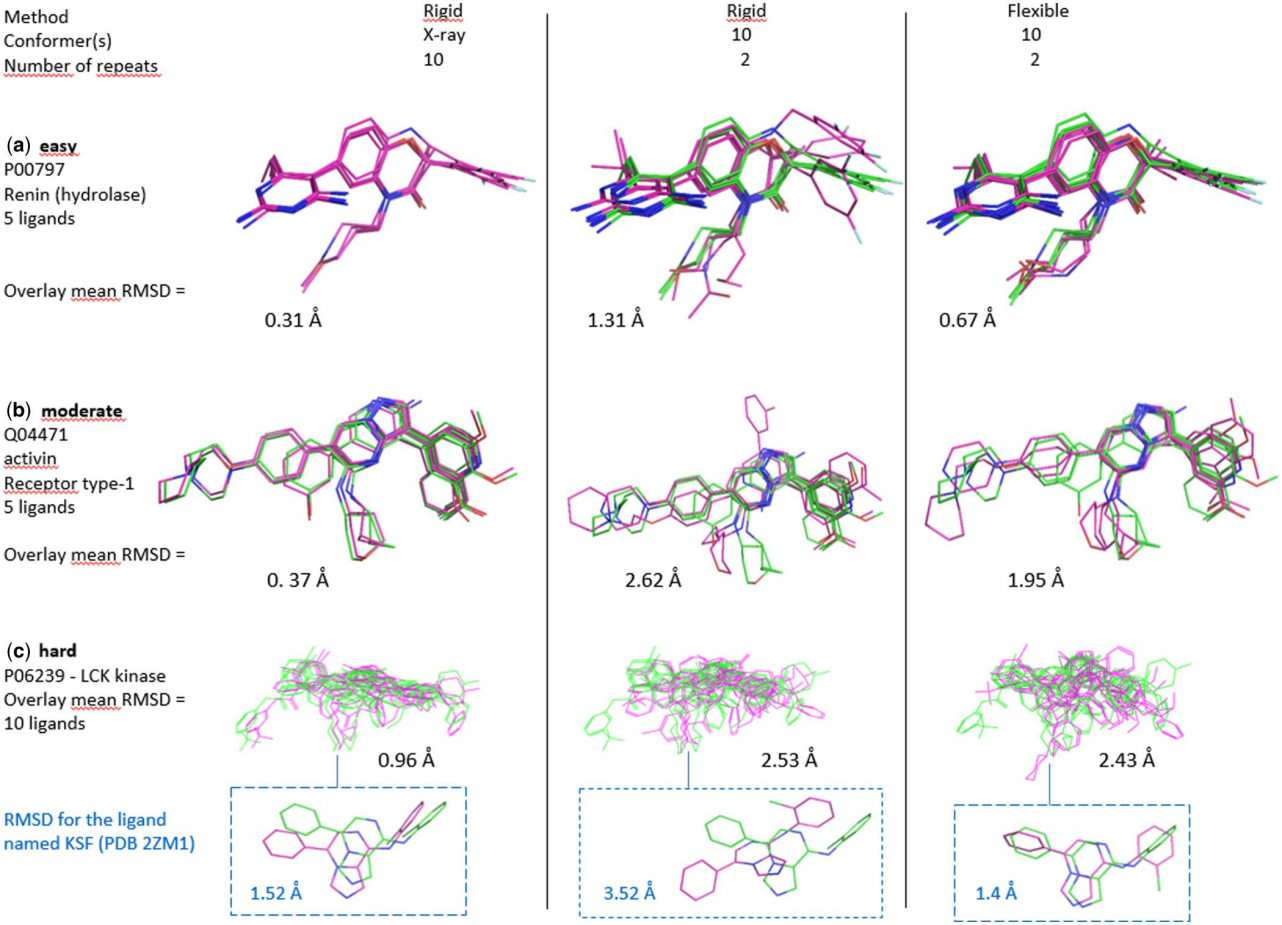
Cross-alignment experiments**—**The X-ray geometry of ligands is in green and predicted pose is in magenta. (a) Easy category—the predicted overlay for P00797 aligns with the experimental one with a mean RMSD value of 0.31 Å when the X-ray conformer is used and a value of 1.31 Å (rigid alignment) and 0.67 Å (flexible alignment) when conformers are employed. (b) Moderate category—the overlay Q04471 is reproduced with a mean RMSD value of 0.37 Å when the X-ray conformer is used and a value of 2.62 Å (rigid alignment) and 1.95 Å (flexible alignment) when conformers are utilized. (c) The overlay P06239 (LCK kinase) belongs to the hard category. As expected, better results are obtained with the X-ray conformer (mean RMSD value of 0.96 Å) than for conformers [2.53 Å (rigid alignment) and 2.43 Å (flexible alignment)]. For visualization purpose, the blue inset displays the superimposition of only one ligand named KSF extracted from PDB 2ZM1. PyMOL version 1.3 was used to generate illustrations.

We first compared the rigid alignments proposed by SENSAAS and the flexible ones proposed by SENSAAS-Flex. Figure 6 shows results in each category of the AZ dataset according to different settings. As expected, results obtained with SENSAAS and the experimental X-ray conformer are globally better whatever the category. However, SENSAAS-Flex significantly outperforms SENSAAS when conformers are utilized. Comparable results are reported in Supplementary Fig. S8. We show that the average RMSD value obtained with SENSAAS-Flex (1.64 Å) is better than the one obtained with SENSAAS (2.10 Å) but worse than the average RMSD value of 0.97 Å when the X-ray conformer itself is aligned. Conformers generated by CORINA lead to comparable results to those of RDKit in flexible alignments although slightly worse (mean RMSD value of 1.69 Å instead of 1.64 Å), a trend already observed in self-alignment experiments.

Whatever the settings, results are related to the degree of difficulty. For instance, the mean RMSD values are 1.13 Å for the easy class, 1.46 Å for the moderate class, 2.32 Å for the hard class and 3.08 Å for the unfeasible class when alignments are performed with SENSAAS-Flex and RDKit’s conformers. The same trend is observed for the three other settings (Supplementary Fig. S8).

In Table 3, we report those results and compare them with the performance of state-of-the-art methods, all based on rigid alignments: SHAFTS (Liu *et al.* 2011), ShaEP (Vainio *et al.* 2009), the Cambridge Structural Database (CSD)-driven overlay program (Giangreco *et al.* 2014), MolAlign (Chan 2017), and PharmScreen (Vázquez *et al.* 2018). SHAFTS, ShaEP, and PharmScreen experiments were performed using X-ray conformations whereas the CSD-driven overlay program and MolAlign used pre-generated conformers. Using the X-ray conformation in cross-alignments allows to only evaluate the performance of the method without handling the conformational flexibility of ligands. RMSD values are not available for the pharmacophore-based experiments from Giangreco et al. (2014), MolAlign, or PharmScreen but, percentage of conformations can be compared (Table 3). Of note, total success, i.e. 100% is impossible because the unfeasible category contains overlays with ligands that are not superimposed (they bind at different sites of the binding pocket as displayed in Supplementary Fig. S2).

**Table 3.** Cross-alignment experiments—Comparison of method performance on the AZ dataset: rigid and flexible alignments with SENSAAS and SENSAAS-Flex, rigid alignments using X-ray or pre-generated conformers with shape-based methods (SHAFTS and ShaEP), pharmacophore-based methods [CSD-driven overlay program published by Giangreco *et al.* (2014) and MolAlign] and the molecular field-based method PharmScreen.^a^

	Molecular Field	Shape-based						Pharmacophore-based	
	**PharmScreen (** Vázquez *et al.* 2018 **)**	SHAFTS	ShaEP	SENSAAS	SENSAAS-Flex	SENSAAS-Flex	SENSAAS	**CSD-driven overlay program (** Giangreco *et al.* 2014 **)**	**MolAlign (** Chan 2017 **)**
Method	Rigid	Rigid	Rigid	Rigid	Flexible	Flexible	Rigid	Rigid	Rigid
Conformers	X-ray	X-ray	X-ray	X-ray	10	10	10	200	average number
Method to generate conformers					RDKit	CORINA	RDKit	^b^	[Balloon (∼60) or Confect (∼27)]
Run(s)	ND	1	1	10	2	2	2	10	5
**Mean RMSD (all)**	ND	**1.09**	**1.06**	**0.97**	**1.64**	**1.69**	**2.10**	ND	ND
Easy set	ND	0.46	0.5	0.37	1.13	1.21	1.78	ND	ND
Moderate set	ND	0.86	0.88	0.80	1.46	1.52	1.97	ND	ND
Hard set	ND	1.71	1.8	1.65	2.32	2.39	2.56	ND	ND
Unfeasible set	ND	3.47	2.58	2.69	3.08	3.07	3.11	ND	ND
**RMSD range (all): [0–2] Å (%)**	ND	**87**	**89**	**89**	**77.8**	**76.1**	**63.3**	ND	ND
Easy set	94	97.8	96.2	98.9	90.8	93	75.1	95	95^c^
Moderate set	79	93	93.1	93.5	82.8	80.5	65.9	73	68^c^
Hard set	54	75.8	79.4	77.9	61.6	60	55.8	39	44^c^
Unfeasible set	31	31.3	53.5	46.4	34.3	32.3	30.3	0	13^c^

a The mean RMSD value is indicated for each experiment condition and category when available. The values for the total are in bold. The percentage of correctly predicted overlays is indicated for each category. ND, not determined.

b Taylor *et al.* (2014).

c Values reported in Vázquez *et al.* (2018).

Our previous study with SENSAAS (Douguet and Payan 2020) showed that 89% of the 1465 ligands in the AZ dataset can be positioned such that the RMSD value with the experimental X-ray structure is below 2.0 Å when the experimental X-ray conformers were used in rigid alignments. Two other shaped-based method, SHAFTS and ShaEP, also performed well with 87% and 89% of ligands in the range [0–2] Å. The mean RMSD values were 0.97 Å for SENSAAS, 1.09 Å for SHAFTS, and 1.06 Å for ShaEP.

As expected, the percentage value decreases significantly when conformers are used in rigid alignments instead of the X-ray conformation: 63.3% of conformations are in the range [0–2] Å instead of 89% with SENSAAS. However, SENSAAS-Flex can improve the results by up to 77.8% of conformations in the range [0–2] Å. When comparing with others methods using pre-generated conformers by category, SENSAAS-Flex is slightly lower in the easy category [93% from CORINA’s conformer (90.8% from RDKit’s conformer), instead of 95% for the CSD-driven overlay program and MolAlign], but significantly outperforms in moderate and hard categories (82.8% and 61.6% instead of 73% and 39% for the CSD-driven overlay program and 68% and 44% for MolAlign, respectively).

## 4 Discussion

We have described SENSAAS-Flex, an add-on of SENSAAS, that uses colored 3D point-based representation of molecular surfaces for performing flexible alignments. Outputs include the superimposition of shapes and 3D graphs as well as similarity scores. In order to optimize the conformation of an aligned structure, SENSAAS-Flex splits the molecular surface and 3D graph to locally optimize the alignment of molecular fragments and sub-shapes before reassembling the structure. This technique is built on concepts of incremental construction algorithms used in docking programs such as FlexX (Rarey *et al.* 1996) where ligands are split into fragments and then reconstructed under the constraint of the binding pocket. These approaches have been found efficient for sampling the conformational space (Rarey *et al.* 1997). SENSAAS-Flex integrates an energy minimization step based on the MMFF94 force field to obtain a low-energy conformation rather than enumerating discrete set of preferred torsion angles as in FlexX. We found that the average difference between the conformational energy of the experimental conformation and the conformation obtained by SENSAAS-Flex is −44 kcal/mol for the AZ dataset in self-alignment experiment (difference ranges between −389 and 2 kcal/mol).

We reported two benchmark studies where the objective was to reproduce the crystal structure geometry of ligands. In self-alignment experiments, we found that SENSAAS-Flex reproduced the crystal structure of the AZ dataset ligands with a mean RMSD value of 0.64 Å when a maximum of ten starting conformers was chosen. In total, 97% of the ligands were aligned with an RMSD deviation of less than 2 Å. Similar nearly perfect matches were obtained with the larger PDBbind refined dataset (mean RMSD value of 0.69 Å and 95% having an RMSD of less than 2 Å). Those results were significantly better than those obtained with rigid alignments only, even when one hundred conformers were taken as input (AZ dataset: mean RMSD value of 1.4 Å and 78% having a RMSD of less than 2 Å).

Cross-alignment experiments have proven to be much more difficult than self-alignments and therefore allow a more accurate assessment of methods. The results of SENSAAS-Flex were slightly lower in the easy category (90.8%) than those of the CSD-driven overlay program and MolAlign (95%) when the conformers were used. However, better performance of SENSAAS-Flex was observed in the moderate and hard categories, these two groups accounting for 1181 out of 1465 ligands (80%).

Our best results were achieved when a small set of different conformers were used as input and when two calculations were executed. Indeed, SENSAAS belongs to stochastic methods and thus, it can produce different results for distinct runs. In addition, we found that the closest conformation from the ensemble of conformers does not automatically lead to the best final conformer. The combination of both parameters allows the generation of different rigid alignments and as many initial poses to optimize. It facilitates the sampling of the conformational space. According to our results, a maximum number of 10 conformers is appropriate to keep a good accuracy and an acceptable running time. We previously showed that SENSAAS (Douguet and Payan 2020) is 10 times slower than popular methods using atom-centered Gaussian functions (Vainio *et al.* 2009, Liu *et al.* 2011) but the ability of our flexible method to find correct geometries with a small set of conformers makes it particularly efficient in that task. In comparison, the ReFlex3D algorithm based on the program ROCS (OpenEye Scientific, Cadence Molecular Sciences) starts with the rigid shape alignment of a number of conformers up to 100 000 before refining the 10 best scored poses (Schmidt *et al.* 2018). SENSAAS-Flex may significantly contribute the 3D shape similarity methods, one of whose weaknesses was precisely the problem of ligand flexibility (Kumar and Zhang 2018).

Most methods address the pharmacophore modeling and the ligand flexibility separately from the shape screening. A significant advantage of our tool is that shape, pharmacophoric features (by the coloration of surfaces) and flexibility are all considered in a single process. However, this integrated solution comes at a cost in terms of computing time. Presently, the execution speed makes SENSAAS-Flex appropriate for virtual screening of small and medium-sized chemical libraries. An improvement may come from our way to sample the conformational space. Future work might investigate the use of a torsion angle database to generate the most relevant conformer by only selecting optimal torsion angle between fragments to link (Guba *et al.* 2015). The use of Python threads or multiprocessing would also give substantial improvement in computation times.

## 5 Conclusion

In the present study, we have described SENSAAS-Flex, an add-on of SENSAAS, for performing flexible molecular alignments. Our results show that 3D point-based representation of surfaces can be employed as a shape constraint to sample the conformational space. A particularly interesting feature of our approach is that it optimizes the superimposition of two molecules by matching similar exposing areas in terms of geometry and properties instead of maximizing the volume overlap as do methods using atom-centered Gaussian functions. This property allows our tools to identify global and local similarities and align structures, substructures, and small fragments.

## Supplementary Material

btae105_Supplementary_Data
