# Supplementary material for: SENSAAS-Flex: a joint optimization approach for aligning 3D shapes and exploring the molecular conformation space
Source: Bioinformatics. 2024 Feb 21;40(3):btae105. doi: 10.1093/bioinformatics/btae105 (PMC10918633; doi:10.1093/bioinformatics/btae105)
Supplement: btae105_Supplementary_Data [file btae105_supplementary_data.pdf]

Supplementary Information for:

SENSAAS-Flex: a joint optimization approach for aligning 3D shapes and exploring the molecular conformation space

Hamza Biyuzan[1], Mohamed-Akram Masrour[1], Lucas Grandmougin[1], Frédéric Payan[1,±] and Dominique Douguet[2,±,]*

[1]Université Côte d'Azur, CNRS, I3S, Les Algorithmes - Euclide B, 2000 route des lucioles 06900 Sophia Antipolis, France

[2]Université Côte d'Azur, Inserm U1323, CNRS UMR 7275, IPMC, 660 route des lucioles 06560 Valbonne, France

[±] Both authors are equivalent last authors

[*] To whom correspondence should be addressed

Contact: douguet@ipmc.cnrs.fr

Supplementary Figure S1

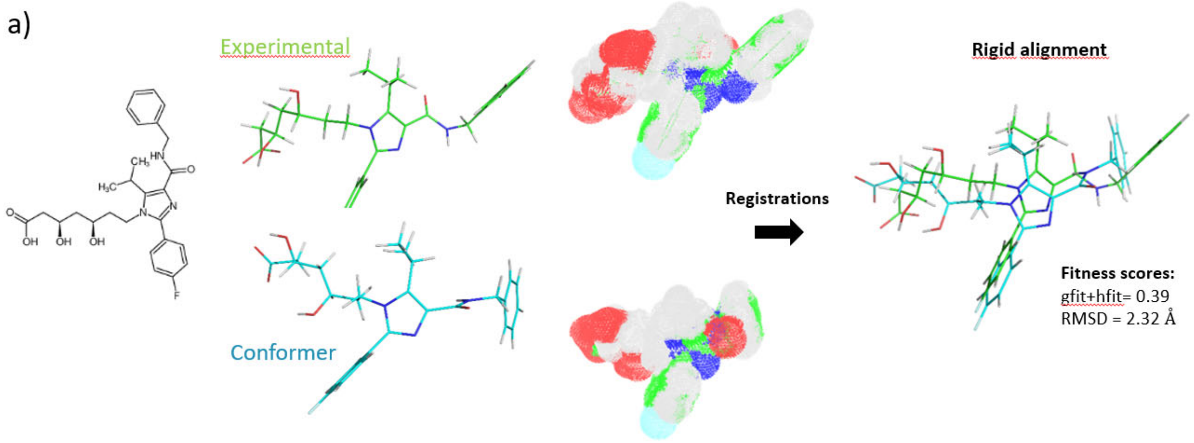

b)

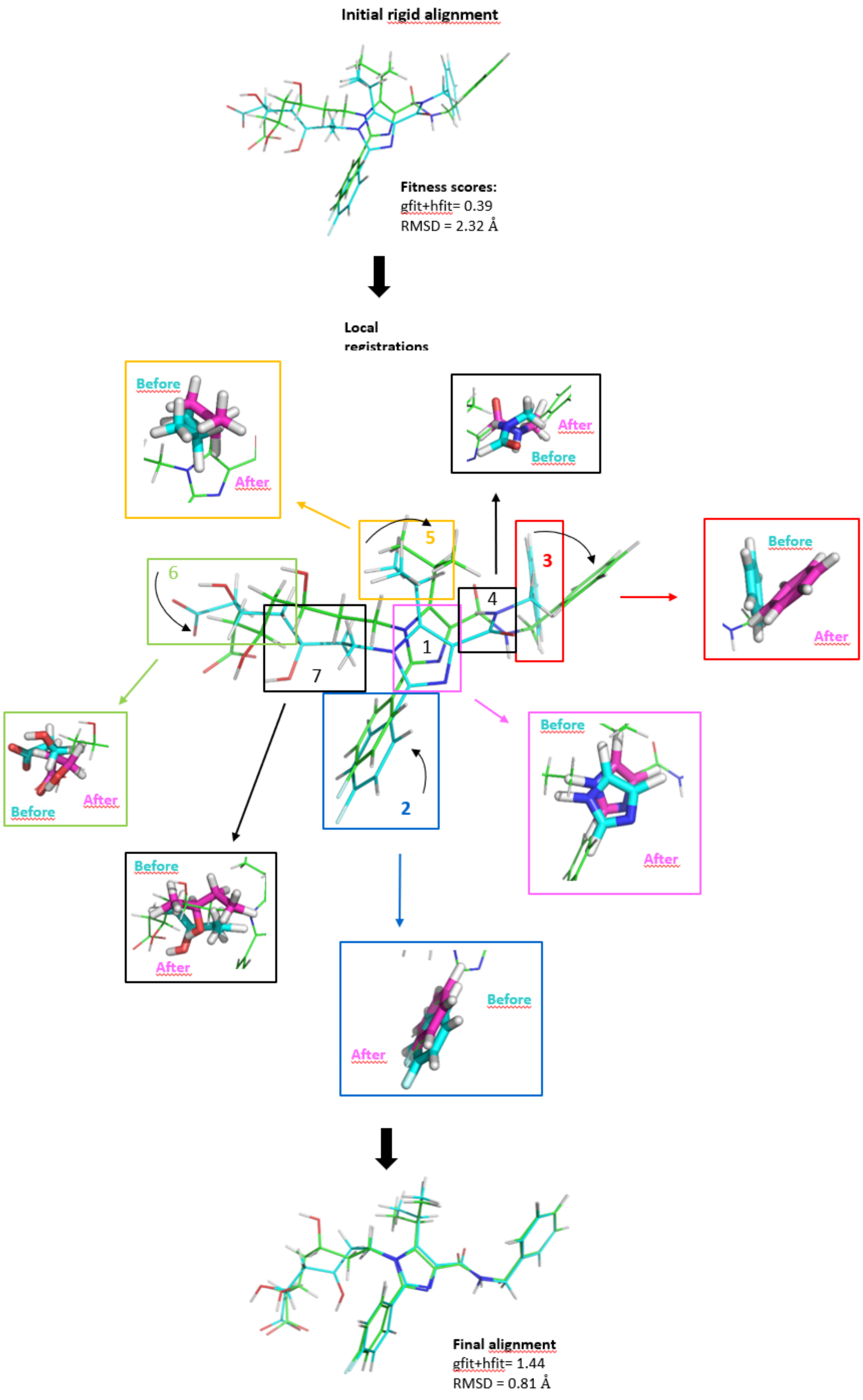
Initial rigid alignment
Fitness scores:
gfit+hfit= 0.39
RMSD = 2.32 Å
Local registrations
Before
After
1
2
3
4
5
6
7
Final alignment
gfit+hfit= 1.44
RMSD = 0.81 Å

Figure S1 Flexible alignment of a ligand with several rotatable bonds. The Target inhibitor 4HI (in green) was extracted from the PDB complex 3CCW (3-hydroxy-3-methylglutaryl coenzyme A reductase (HMGR); Uniprot P04035). The structure of the Source in cyan is a conformer of 4HI generated using RDKit. a) Initial rigid alignment generated using SENSAAS. b) SENSAAS-Flex aims at improving the alignment of each of the seven fragments of 4HI according to the neighboring Target shape (the 3D point-based representation of the Target shape is not shown for visualization purpose). Initial and final positions of fragments are shown in cyan and magenta, respectively. The superimposition, final fitness score (gfit+hfit = 1.44) and RMSD value of 0.81 Å indicate a significant improvement of the initial alignment (fitness score gfit+hfit of 0.39 and RMSD value of 2.32 Å).

Supplementary Figure S2

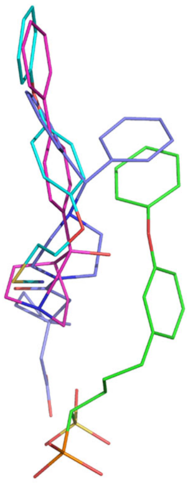

Figure S2 Example of the unfeasible overlay A9JQL9 (dehydrosqualene synthase). The ligand in green does not overlap the others. In such case, the total success of a cross-alignment experiment is impossible.

Supplementary Figure S3

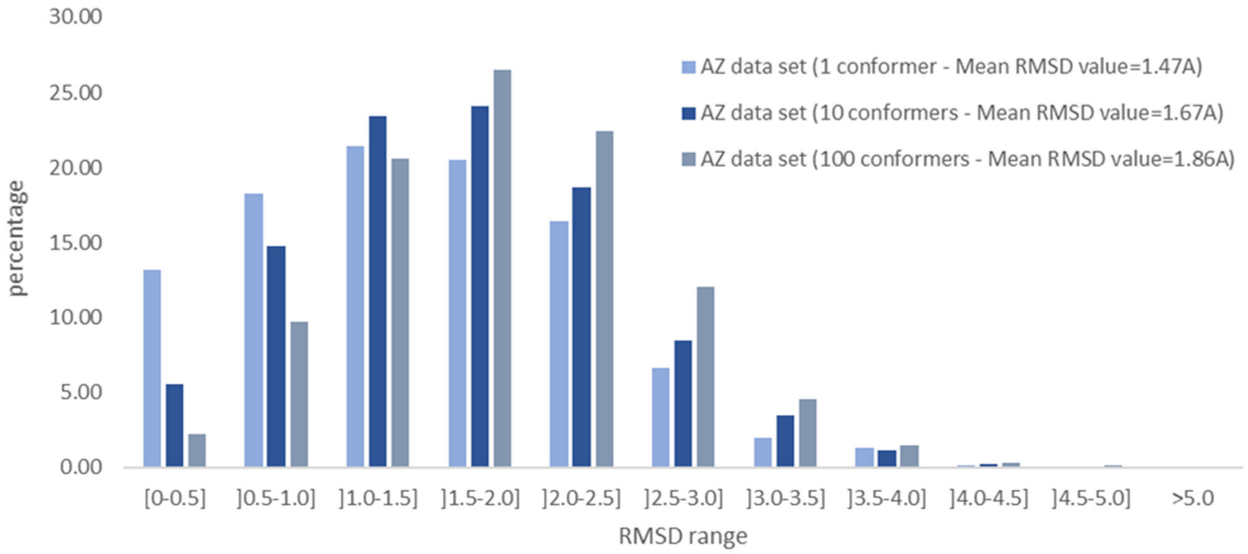

Figure S3 Distribution of RMSD values for all conformers in the AZ data set when RDKit was used to generate one, ten or one hundred conformers.

Supplementary Figure S4

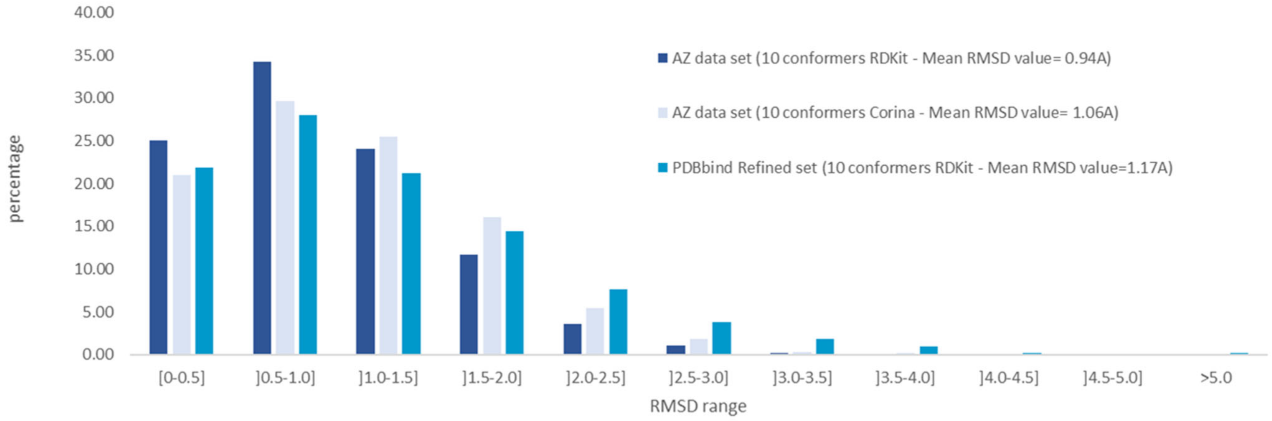

Figure S4 Distribution of RMSD values for the closest conformers in the AZ data set when RDKit or CORINA was used to generate ten conformers and RMSD values for the closest conformers for the PDBbind refined data set when RDKit was used to generate ten conformers.

Supplementary Figure S5

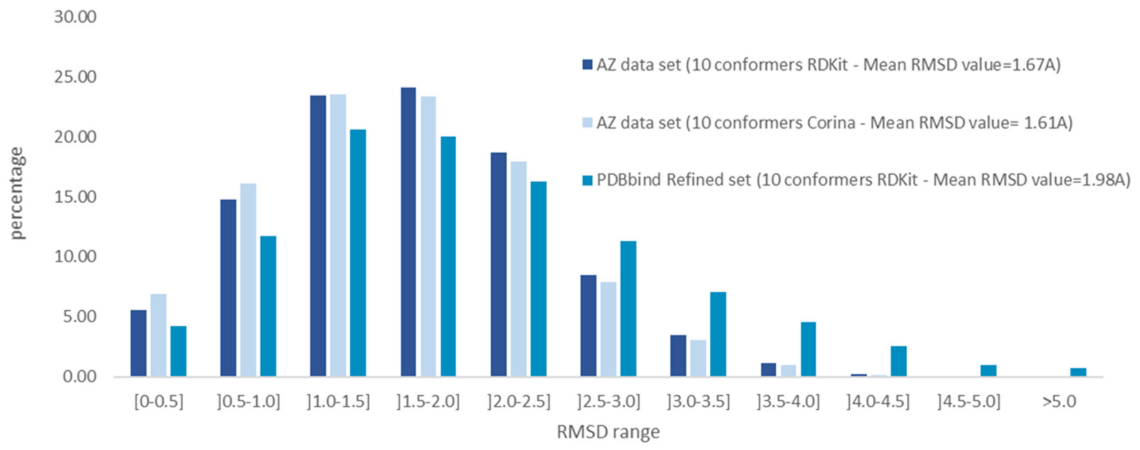

Figure S5 Distribution of RMSD values for all conformers in the AZ data set when RDKit or CORINA was used to generate ten conformers and RMSD values for all conformers in the PDBbind refined data set when RDKit was used to generate ten conformers.

Supplementary Figure S6

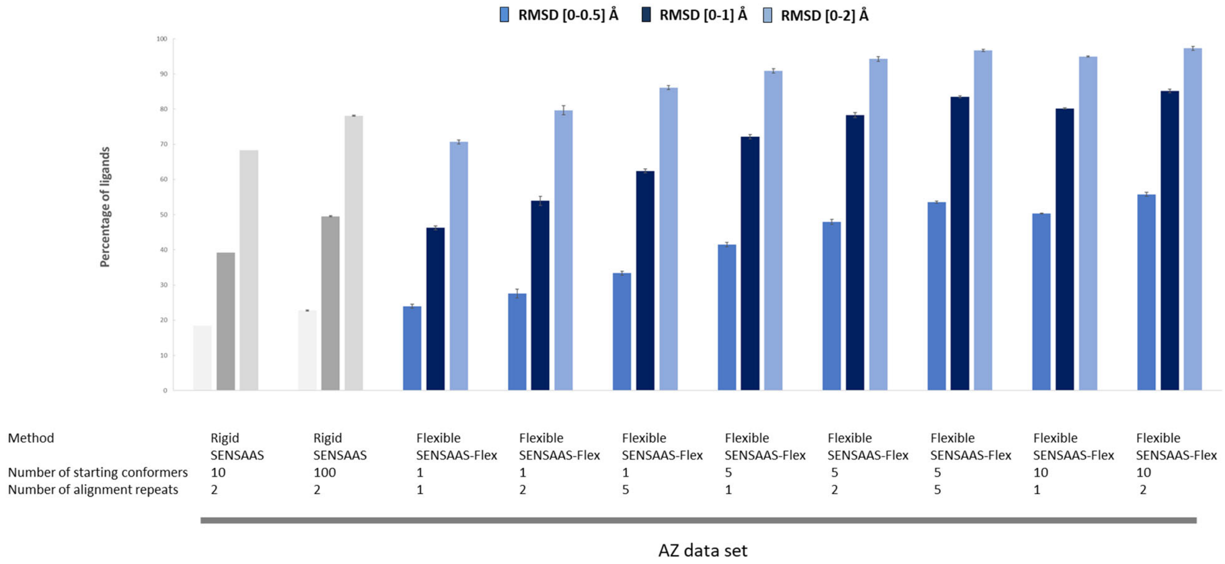

Figure S6 Self-alignment experiments using SENSAAS and SENSAAS-Flex - Mean RMSD values of rigid and flexible alignments in function of the number of starting conformations and runs. Histograms indicate the percentage of ligands showing a structural deviation between the aligned conformer and the experimental X-ray structure of less than 0.5 Å (first bar), 1 Å (second bar) and 2 Å (third bar). A standard deviation value was calculated over 5 experiments.

Supplementary Table S1

Table S1. Self-alignment experiment using SENSAAS and SENSAAS-Flex - Rigid and flexible alignment results in function of the conformer generator. The mean RMSD value and percentage of conformations in three ranges [0-0.5] Å, [0-1] Å and [1-2] Å are indicated for each experiment condition.

	**AZ data set**			
Method	Rigid	Flexible	Rigid	Flexible
Nb	10	10	10	10
conformers	RDKit	RDKit	CORINA	CORINA
Run(s)	2	2	2	2
Mean RMSD	1.77	0.64	1.94	0.76
RMSD range:				
[0-0.5] Å (%)	18.4	55.8	16.3	48.9
[0-1] Å (%)	39.2	85.1	34.8	79.9
[0-2] Å (%)	68.3	97.3	62.1	94.9

Supplementary Figure S7

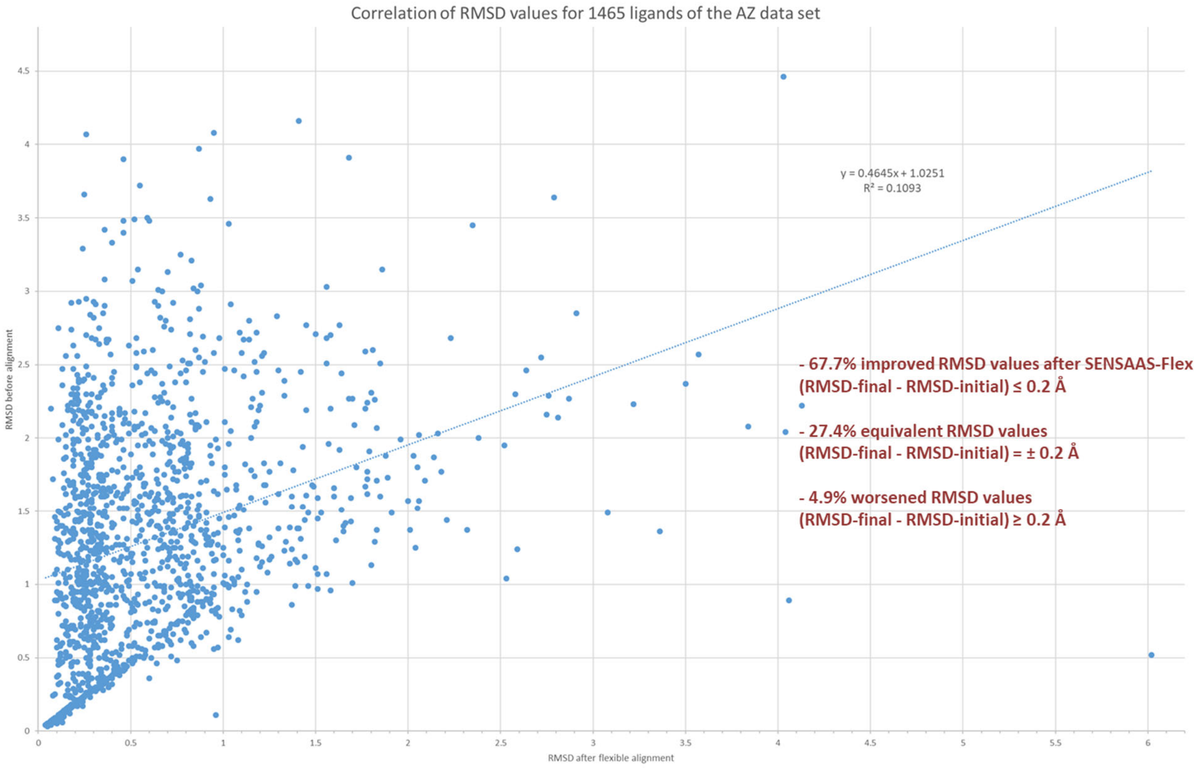

Figure S7 Self-alignment experiment - Correlation between the RMSD value of the conformer before and after the alignment with SENSAAS-flex for one experiment using ten starting conformations and two runs. A simple linear regression gives the following equation Y = 0.4645 X + 1.0251 with a correlation coefficient R^2 = 0.1093. The analysis shows that SENSAAS-Flex improves the RMSD value for 67.7% of initial conformers, does not change the RMSD value for 27.4% and worsens the RMSD value for 4.9%.

Supplementary Figure S8

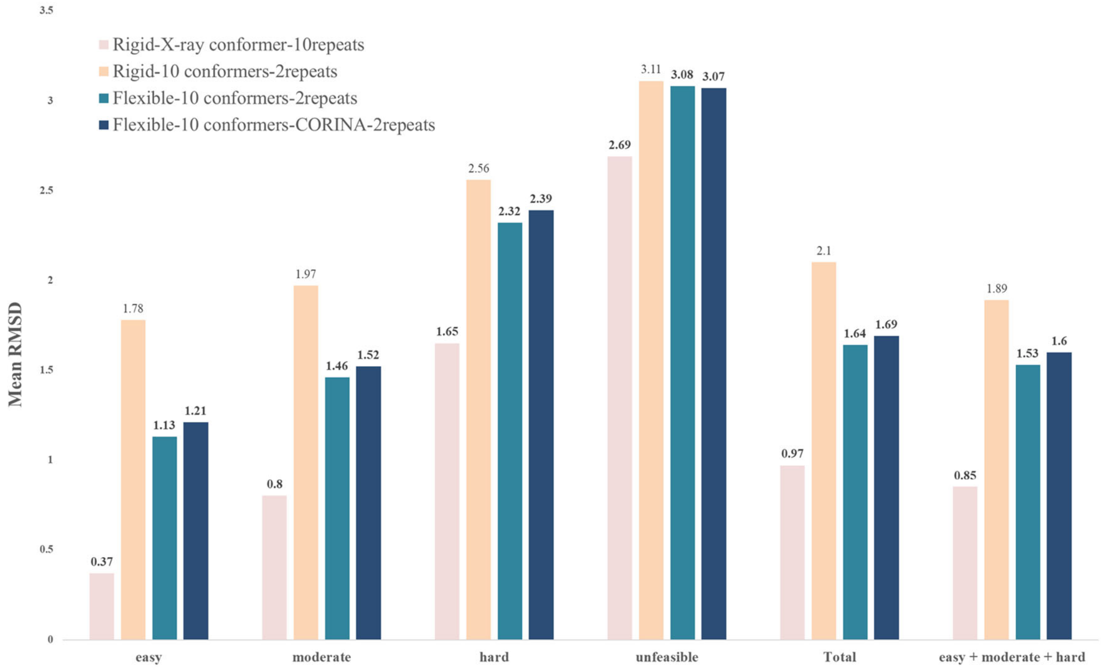

Number of ligands:	185	991	190	99	1465	1366

Figure S8 Cross-alignment experiments - Mean RMSD values of rigid and flexible alignments with the AZ data set in function of the starting conformation(s) (X-ray, ensemble of conformers using RDKit (default) or CORINA). Histograms indicate the mean RMSD value for each category in function of the experiment conditions.
